# LRP1 Regulates Architecture of the Vascular Wall by Controlling PDGFRβ-Dependent Phosphatidylinositol 3-Kinase Activation

**DOI:** 10.1371/journal.pone.0006922

**Published:** 2009-09-09

**Authors:** Li Zhou, Yoshiharu Takayama, Philippe Boucher, Michelle D. Tallquist, Joachim Herz

**Affiliations:** 1 Department of Molecular Genetics, UT Southwestern Medical Center, Dallas, Texas, United States of America; 2 Molecular Biology, UT Southwestern Medical Center, Dallas, Texas, United States of America; 3 Department of Pharmacology, University of Strasbourg, Strasbourg, France; Monash University, Australia

## Abstract

**Background:**

Low density lipoprotein receptor-related protein 1 (LRP1) protects against atherosclerosis by regulating the activation of platelet-derived growth factor receptor β (PDGFRβ) in vascular smooth muscle cells (SMCs). Activated PDGFRβ undergoes tyrosine phosphorylation and subsequently interacts with various signaling molecules, including phosphatidylinositol 3-kinase (PI3K), which binds to the phosphorylated tyrosine 739/750 residues in mice, and thus regulates actin polymerization and cell movement.

**Methods and Principal Findings:**

In this study, we found disorganized actin in the form of membrane ruffling and enhanced cell migration in LRP1-deficient (*LRP1−/−*) SMCs. Marfan syndrome-like phenotypes such as tortuous aortas, disrupted elastic layers and abnormally activated transforming growth factor β (TGFβ) signaling are present in smooth muscle-specific LRP1 knockout (*smLRP1−/−*) mice. To investigate the role of LRP1-regulated PI3K activation by PDGFRβ in atherogenesis, we generated a strain of *smLRP1−/−* mice in which tyrosine 739/750 of the PDGFRβ had been mutated to phenylalanines (PDGFRβ F2/F2). Spontaneous atherosclerosis was significantly reduced in the absence of hypercholesterolemia in these mice compared to *smLRP1−/−* animals that express wild type PDGFR. Normal actin organization was restored and spontaneous SMC migration as well as PDGF-BB-induced chemotaxis was dramatically reduced, despite continued overactivation of TGFβ signaling, as indicated by high levels of nuclear phospho-Smad2.

**Conclusions and Significance:**

Our data suggest that LRP1 regulates actin organization and cell migration by controlling PDGFRβ-dependent activation of PI3K. TGFβ activation alone is not sufficient for the expression of the Marfan-like vascular phenotype. Thus, regulation of PI3 Kinase by PDGFRβ is essential for maintaining vascular integrity, and for the prevention of atherosclerosis as well as Marfan syndrome.

## Introduction

Low density lipoprotein receptor related protein 1 (LRP1) is a multifunctional member of the LDL receptor (LDLR) gene family with a unique capacity of binding over 40 distinct ligands [1]. It plays diverse roles in a variety of biological processes including lipoprotein metabolism, protease degradation, activation of lysosomal enzymes, and endocytosis of bacterial toxins and viruses [1], [2]. Binding of apolipoprotein E (apoE) to the extracellular domain of LRP1 removes apoE-containing lipoprotein remnants from the circulation into the liver by endocytosis [3], [4], [5]. By contrast, in the smooth muscle cells (SMCs) of the arterial wall, apoE-lipoprotein binding inhibits platelet-derived growth factor (PDGF)-directed SMC migration [6]. Studies from our laboratory have shown that LRP1 suppresses PDGF receptor β (PDGFRβ) activation and protects against atherosclerosis [7].

Activated PDGFRβ undergoes tyrosine phosphorylation and subsequently interacts with a variety of SH2 domain-containing signaling molecules including phosphatidylinositol 3-kinase (PI3K), phospholipase Cγ (PLCγ), Src family kinase, and phosphotyrosine phosphatase SHP-2 [8]. Among these interacting proteins, PI3K which binds to the phosphorylated tyrosine 740/751 residues (739/750 in the mouse) of PDGFRβ through its p85 regulatory subunit [9], is particularly important for regulating actin organization [10], [11], cell growth and migration [12].

LRP1 is also known as transforming growth factor β (TGFβ) receptor V (TβR-V) and appears to be required for mediating the growth inhibitory response of TGFβ, in conjunction with Smad signaling through TβR-II and I [13], [14]. TGFβ signaling is abnormally elevated in the absence of LRP1 *in vivo*, where analysis of SMC-specific LRP1 knockout (smLRP1−/−) mice revealed a Marfan syndrome-like phenotype with nuclear accumulation of phosphorylated Smad2 (p-Smad2) and disruption of elastic layers in the vessel wall [15].

For the present study we have generated a new, genetically complex strain of compound mutant mice that are LDL receptor-deficient (LDLR−/−), lack LRP1 only in their vascular smooth muscle cells, and express an endogenous, crippled form of the PDGFRβ that is incapable of activating PI3K. Our goal was to test, whether increased PDGFRβ signaling through PI3K is the primary cause for the increased susceptibility to atherosclerotic lesion development in LDLR −/− mice lacking LRP1 in their SMCs, and whether PDGFRβ-dependent PI3K signaling is required for the expression of the Marfan syndrome-like phenotype in smLRP1-deficient mice.

## Results

### Lack of LRP1 expression in the SMCs results in cell hypertrophy and vessel elongation

Earlier data from our laboratory showed that sm*LRP1*−/−; *LDLR*−/− mice are highly susceptible to atherosclerosis when fed a high-cholesterol diet [7]. To determine if this increased susceptibility to atherosclerosis is preserved in sm*LRP1*−/− mice in the absence of hypercholesterolemia, sm*LRP1*−/− mice either expressing or lacking LDLR were maintained on a standard low-fat rodent chow. Determination of plasma total cholesterol confirmed that sm*LRP1*−/− mice did not develop hypercholesterolemia (129.9±5.3 mg/dl, Table 1). Whereas, sm*LRP1*−/−; *LDLR*−/− mice had high total cholesterol levels of 246.5±61.0 mg/dl with a major increase in LDL (Figure S1).

**Table 1:** Total cholesterol and triglyceride levels of mouse plasma.

	WT	smLRP1−/−	LDLR−/−	smLRP1−/−, LDLR −/−
**Total Cholesterol**	131.3±16.8	129.9±5.3 a	294.2±17.7 b	246.5±61.0c,d
**Total Triglycerides**	61.7±9.8	51.1±20.0 a	125.0±42.9 b	101.1±28.4 c,d

Total cholesterol and triglyceride levels were significantly higher in mice lacking LDLR, even though they were fed a standard rodent chow diet. LRP-deficient in smooth muscle cells did not change the lipid levels significantly.

a *p*>0.05 (*smLRP1−/−* vs *WT*).

b *p*<0.01 (*LDLR−/−* vs *WT*).

c *p*<0.05 (*smLRP1−/−; LDLR−/−* vs *smLRP1−/−*).

d *p*>0.05 (*smLRP1−/−; LDLR−/−* vs *LDLR−/−*).

**Figure 1:**
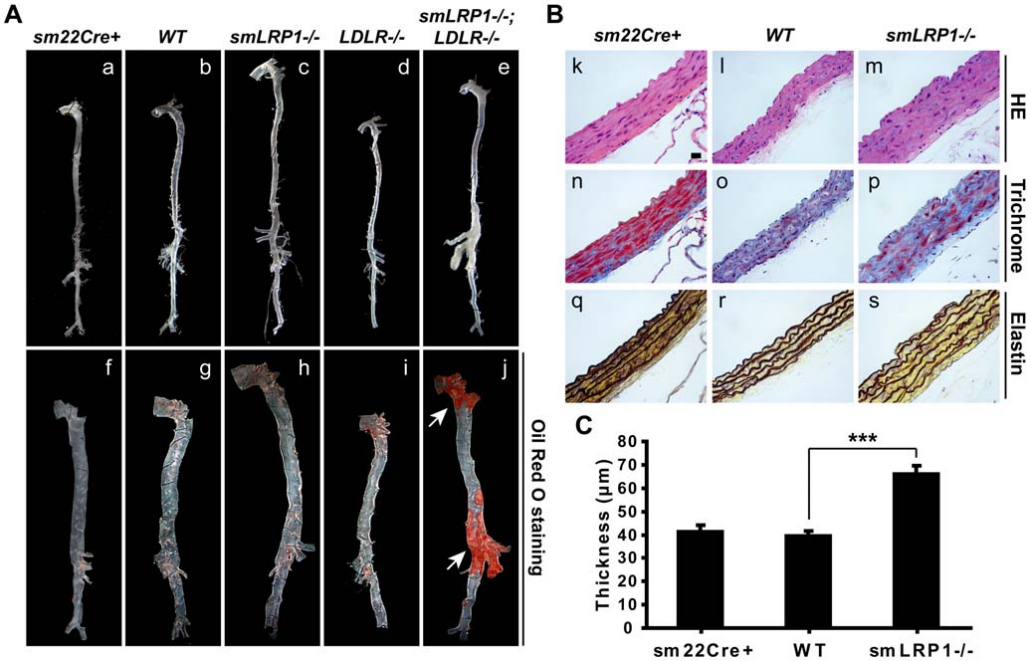
Hypertrophic and hyperplastic SMCs and elongated aortas in *smLRP1−/−* mice. (A) Unopened (a–e) and Oil Red O stained (f–j) aortas from 11-month old mice of the indicated genotypes. Mice were maintained on standard rodent chow diet. Arrows indicate lipid-laden atherosclerotic lesions. (B) Histological analysis of thoracic aortas from 11-month old mice of the indicated genotypes. k–m: hematoxylin & eosin (HE); n–p: trichrome; q–s: elastin staining. Scale bar, 20 µm. (C) Thickness of the aortas of the indicated genotypes was quantified using Image J software (NIH). Data represent mean±SD from 3 mice per group. *** *p*<0.001.

Elongated aortas were present in the absence of hypercholesterolemia in sm*LRP1*−/− mice (Figure 1c, h). However, atherosclerotic lesions were only visible in sm*LRP1*−/−; *LDLR*−/− mice (Figure 1e, j). To study structural changes in the vascular wall of these elongated aortas, H&E, trichrome and elastin staining were performed. Thickened aortic walls with intima thickening, disarranged and hypertrophic SMCs, increased extracellular collagen accumulation, and elastic lamina disruption were observed in 11-month old sm*LRP1*−/− mice (Figure 1B). Vascular wall thickening was also present in young mice at 7 weeks of age (Figure S2). Compared with wild type, the aortic wall of sm*LRP1*−/− mice was significantly thicker (66.14±3.32 µm *vs.* 39.35±2.16 µm, Figure 1C). Our findings indicate that LRP1 expression in SMCs controls the architecture of the vascular wall in a plasma cholesterol-independent manner.

### LRP1 controls PI3K binding and activation by PDGFRβ *in vivo*

**Figure 2:**
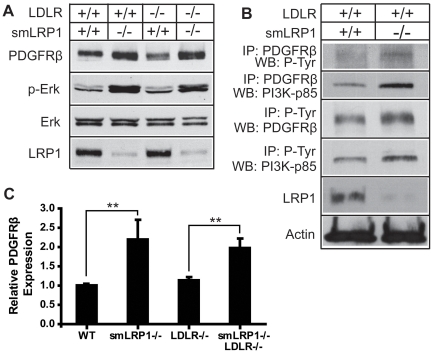
Increased expression and activation of PDGFRβ and PI3K binding by PDGFRβ in aortic extracts of *smLRP1−/−* mice. (A) Protein extracts (10 µg/lane) from mouse aortas of the indicated genotypes were analyzed by immunoblotting for PDGFRβ, p-Erk1/2, Erk1/2, and LRP1. (B) 200 µg of aortic extracts of the indicated genotypes were immunoprecipitated with the designated antibodies (anti-PDGFRβ and anti-phosphotyrosine) to semi-quantitatively determine the interaction between PI3K and PDGFRβ. Precipitated proteins were analyzed by immunoblotting using the indicated antibodies (anti-phosphotyrosine, anti-PI3K-p85 and anti-PDGFRβ). Actin served as a loading control. WB: Western blot; IP: immunoprecipitation. (C) Expression of PDGFRβ relative to the loading control was quantified using Image J software (NIH). Data are expressed as mean±SD. ** *p*<0.01.

PDGFRβ signaling is crucial for regulating SMC responses and is an important contributing factor to atherogenesis. Previous work from our laboratory showed dramatically increased expression and activation of PDGFRβ in aortas from sm*LRP1*−/−; *LDLR*−/− mice on a high-cholesterol diet [7]. To investigate the basal expression and activation level of PDGFRβ in mice maintained on a low-cholesterol diet, aortic extracts were prepared from mice expressing or lacking LRP1 and LDLR and analyzed by Western blotting. Because PDGFRβ activation, through transphosphorylation of tyrosine residues in its cytoplasmic domain, triggers a cascade of phosphorylation events which eventually lead to the activation of extracellular regulated-protein kinases (Erks), phosphorylated-Erk1/2 was used as an indicator of PDGFRβ activation [7]. About a two-fold increase of PDGFRβ expression was detected in sm*LRP1*−/− mice regardless of LDLR genotype (Figure 2A, C). Increased Erk1/2 phosphorylation was also observed in these aortas (Figure 2A). These data suggest that the expression and activation of PDGFRβ is only regulated by LRP1, not LDLR.

PI3K binding sites on PDGFRβ are crucial for the receptor-mediated cell responses [16], [17]. To explore the functional and biochemical interaction between LRP1 and the PDGFRβ-PI3K signaling pathway, we performed a co-immunoprecipitation assay. Compared to wild type animals, sm*LRP1*−/− mice showed increased tyrosine-phosphorylation of and PI3K binding to PDGFRβ (Figure 2B), indicating that LRP1 regulates PDGFRβ-dependent activation of PI3K by controlling PDGFRβ phosphorylation.

### Disruption of PDGFRβ-PI3K signaling in mice reduces atherosclerosis

To investigate whether LRP1 regulates atherosclerosis through the PDGFRβ-dependent PI3K pathway *in vivo*, we generated a compound mutant mouse model by crossing *smLRP1*−/−; *LDLR*−/− mice to PI3K binding-deficient *PDGFRβ F2/F2* mutant mice, in which tyrosine residues at position 739 and 750 are mutated to phenylalanines [18].

**Figure 3:**
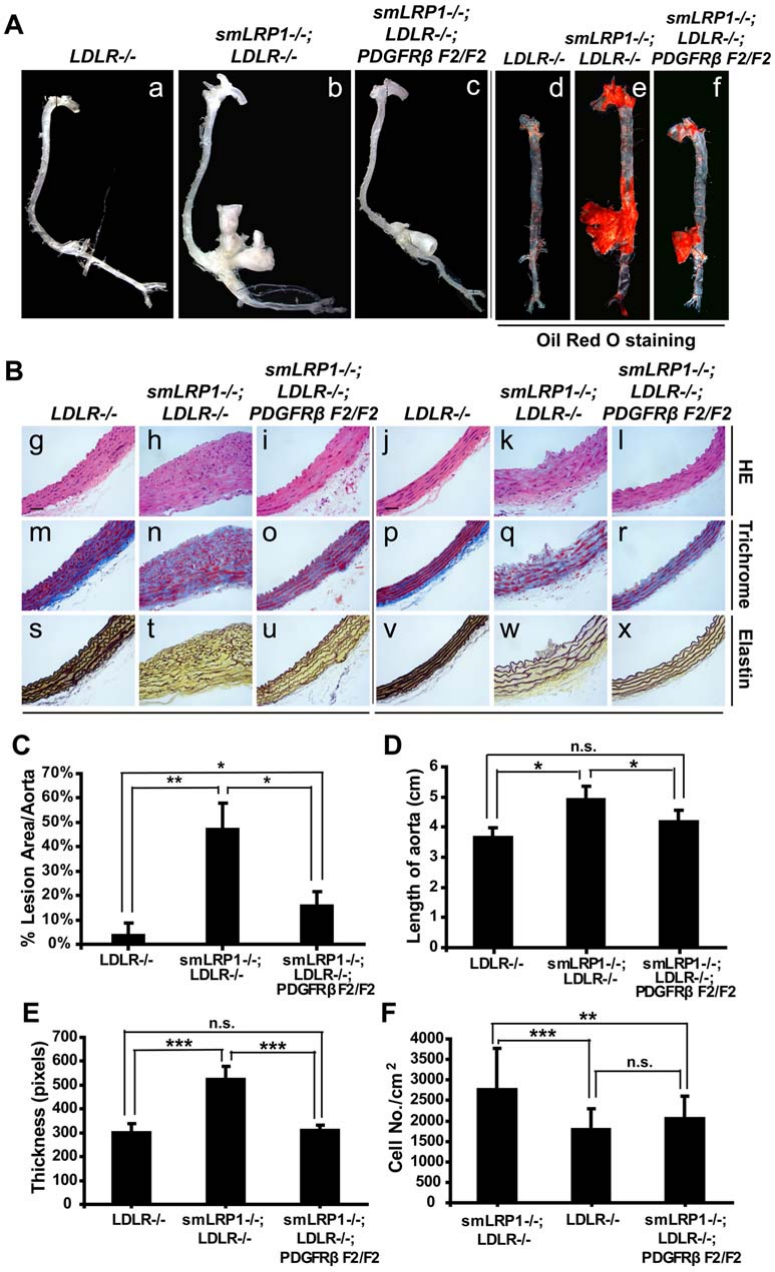
Reduced atherosclerotic lesions in *smLRP1−/−; LDLR−/−; PDGFRβ F2/F2* mutant mice. (A) Unopened (a–c) & Oil Red O stained (d–f) aortas from 10-month old mice of the indicated genotypes. Mice were maintained on standard rodent chow diet. Arrows indicate lipid-laden atherosclerotic lesions. (B) Histological analysis of aortas from 8-month old mice of the indicated genotypes. g–l: HE stain, m–r: trichrome stain, s–x: elastin stain. g,h,i,m,n,o,s,t u: aortic arch; j,k,l,p,q,r,v,w,x: thoracic aorta. Scale bar, 20 µm. (C, D, E, F) Atherosclerotic lesions (C), length (D), thickness (E) and cell number per cm^2^ (F) of the indicated genotypes were quantified using Image J software (NIH). Results from 3 mice per group are presented as mean±SD. * *p*<0.05, ** *p*<0.01, *** *p*<0.001.

We found significantly decreased atherosclerotic lesions in the aortic arch and abdominal aorta of *smLRP1−/−; LDLR−/−; PDGFRβ F2/F2* mice (Figure 3A, C). H&E, trichrome and elastin staining revealed well-arranged spindle-shaped SMCs, reduced extracellular matrix, and virtually normal elastic layers in *smLRP1−/−; LDLR−/−; PDGFRβ F2/F2* aortas (Figure 3B). Vascular wall thickness, hypercellularity and length of the aortas in *smLRP1−/−; LDLR−/−; PDGFRβ F2/F2* mutants were markedly reduced to approximately normal levels (Figure 3B, D, E, F). However, the prominent aneurysms of the mesenteric arteries, which are a hallmark of *smLRP−/−* mice, were notably not abolished in *smLRP1*−/−; *LDLR*−/−; *PDGFRβ F2/F2* mice (Figure 3A), suggesting that atherogenesis and aneurysm formation employ at least partially different molecular or regionally distinct mechanisms.

### Ablation of PI3K binding to PDGFRβ reverses actin disorganization in LRP1-deficient SMCs

**Figure 4:**
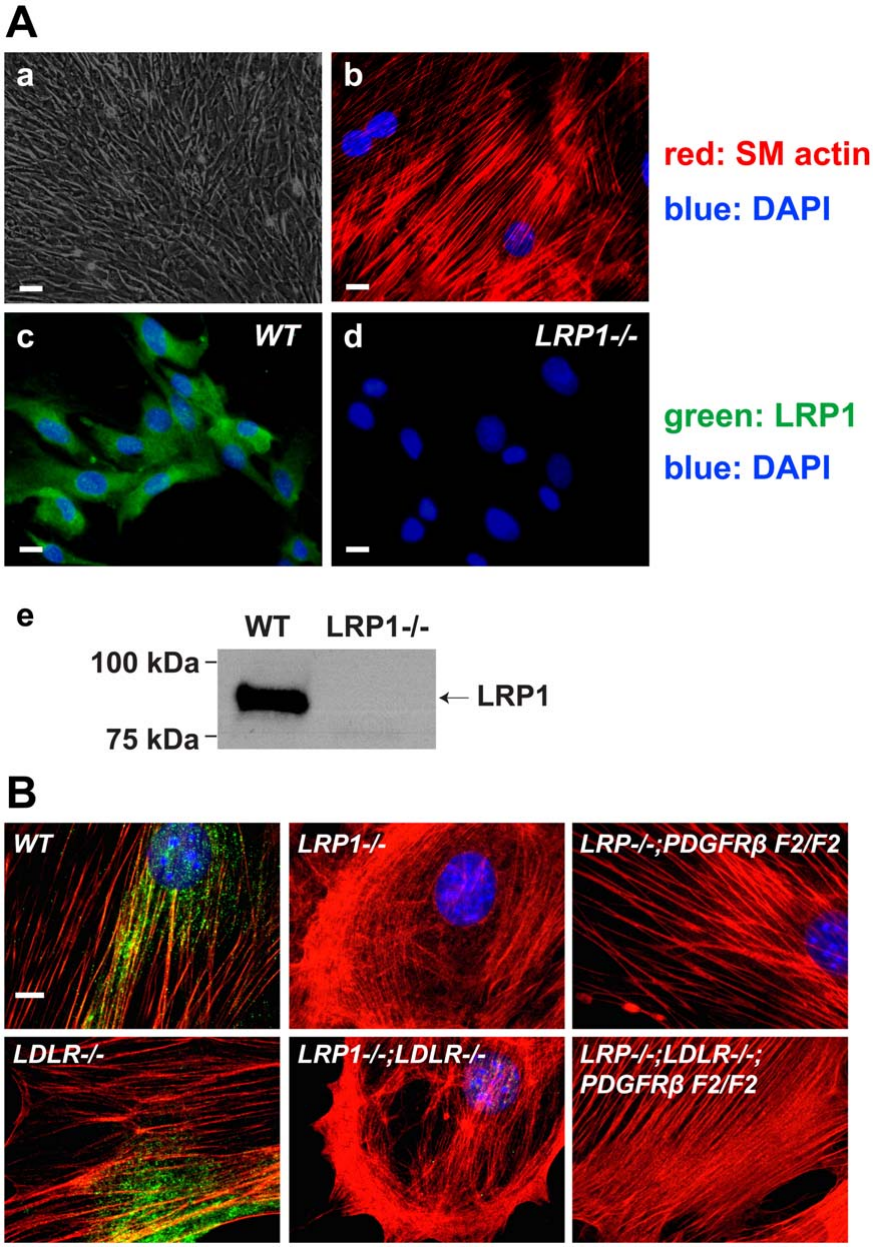
Disorganization of the actin cytoskeleton in *LRP1−/−* SMCs is prevented by blocking PI3K activation by PDGFRβ. (A) Primary SMCs generated from mouse aortas. (a) Phase contrast image. Scale bar, 80 µm. (b) Immunofluorescence using anti-smooth muscle actin monoclonal antibody (red). Blue: DAPI staining of nuclear DNA. Scale bar, 40 µm. (c, d) Detection of LRP1 (green) by immunofluorescence by a rabbit anti-LRP1 polyclonal antibody. Blue: DAPI staining. WT, wild type. Scale bars, 40 µm. (e) Immunoblotting was performed to verify the presence or absence of LRP1 protein in the wild type and *LRP1−/−* SMCs using the same polyclonal anti-LRP1 antibody. (B) Immunofluorescence of smooth muscle actin (red) in primary SMCs. Actin disorganization in *LRP1−/−* and *LRP1−/−; LDLR−/−* SMCs. Normal organization of the actin cytoskeleton is restored in primary *PDGFRβ F2/F2* SMCs. Blue: DAPI. Scale bar: 20 µm.

To characterize SMCs lacking either LRP1 or LDLR and with crippled PI3K binding to PDGFRβ *in vitro*, we generated primary cells from the aortas of these mice. To eliminate fibroblast contamination, LRP1-deficient SMCs were selected with *Pseudomonas* exotoxin A (PEA) according to previous publications [19], [20]. Primary SMCs were identified by their typical spindle shape (Figure 4a) and the expression of smooth muscle actin (Figure 4b). LRP1 expression was verified by both immunocytochemical staining (Figure 4c, d) and Western blotting (Figure 4e). Interestingly, the morphology of SMCs of different genotypes looked quite distinct from each other. Actin organization was disrupted in the SMCs lacking LRP1 expression and an “actin ring” was present below the plasma membrane (Figure 4B). However, in *smLRP1−/−; PDGFRβ F2/F2* SMCs actin organization was restored (Figure 4B), suggesting that LRP1 mediates actin remodeling through PI3K activation upon PDGFRβ phosphorylation.

### Increased migration of LRP1-deficient SMCs is diminished by the PDGFRβ F2 mutation

**Figure 5:**
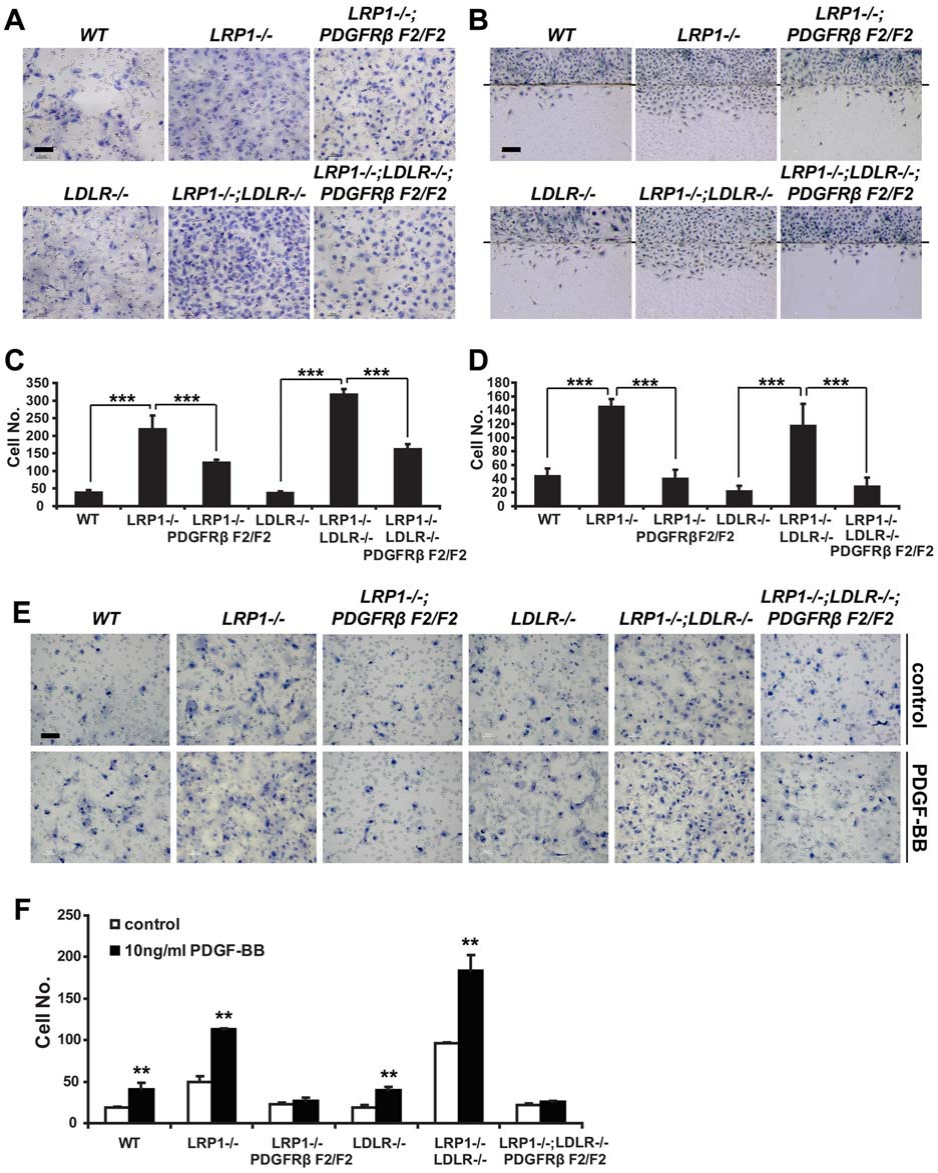
Primary SMC Migration. (A) Transwell migration assay. 30,000 SMCs of the indicated genotypes were added to the top compartment of a Boyden chamber. After 6 hours of incubation, the transwell membrane was fixed and stained with hematoxylin. Cells that had migrated through the holes on the membrane to the bottom face were counted. Scale bar, 50 µm. (B) Scratch assay. 300,000 SMCs of the indicated genotypes were seeded into 60 mm culture dishes in a medium containing 10 µg/ml mitomicin C to prevent cell proliferation and allowed to adhere overnight. The next day, part of the dish was denuded by scratching along a straight line (indicated by a black line behind). Cells were then allowed to migrate into the denuded area for 24 hours prior to fixation and quantification. Scale bar, 50 µm. (C, D) Statistical analyses of the Transwell and Scratch migration assays. Results are represented as mean±SD. *** *p*<0.001. (E) PDGF-BB chemotaxis assay. 10,000 SMCs of the indicated genotypes were added to the top compartment of the Boyden chamber. 10 ng/ml PDGF-BB was added to the lower chamber of the well. After 6 hours of incubation, the transwell membrane was fixed and stained with hematoxylin. The cells that had migrated through the holes on the membrane to the bottom face were counted. Scale bar, 50 µm. (F) Statistical analysis of the PDGF-BB chemotaxis assay. Results are represented as mean±SD. ** *p*<0.01, *** *p*<0.001. n = 5 for all assays.

Our *in vivo* experiments showed disarranged SMCs in the medial layer of the aorta (Figure 1m, 3h, 3k) and disrupted elastic laminas when LRP1 was deficient in the SMCs (Figure 1s, 3t, 3w). To investigate if these phenomena are caused by abnormal migration due to the absence of LRP1, we performed two different kinds of *in vitro* migration assays. Compared with wild type cells, SMCs lacking LRP1 showed markedly increased migratory activity, both in a Boyden chamber transmigration assay (Figure 5A, C) and in the commonly used scratch assay, in which the migration of the cells into a denuded area of a tissue culture dish is quantified (Figure 5B, D). Cell migration was significantly reduced in those cells containing the *PDGFRβ F2/F2* mutation (Figure 5A–D), and this correlated with the improved architecture of the elastic layers in the aortic wall of *smLRP−/−; LDLR−/−; PDGFRβ F2/F2* mice (Figure 3u, x). These findings thus confirm that SMC migration is regulated by LRP1 through the PDGFRβ-dependent PI3K pathway.

### PDGF-BB-induced chemotaxis is inhibited by blocking PI3K activation through PDGFRβ

To investigate whether PDGF-BB-induced SMC migration involves the PDGFRβ-PI3K pathway, 10 ng/ml PDGF-BB was administrated as a chemo-attractant in the bottom well of a Boyden chamber. As shown in Figure 5E, PDGF-BB induced the transmigration of SMCs independent of LRP1 expression. However, when the Y739/750 phosphorylation sites of PDGFRβ were mutated, this PDGF-BB-induced chemotaxis was completely abolished. This finding suggests that the increased migratory propensity of LRP1-deficient SMCs, which is further enhanced by PDGF-BB activation of PDGFRβ, is in its entirety dependent upon the activation of PI3K by PDGFRβ.

### Abnormal activation of TGFβ signaling is present in the absence of LRP1

Our *in vivo* findings indicate that lack of LRP1 in SMCs results in elongation of the aorta, thickening of the vascular wall, and disruption of the elastic layers. Tortuous aorta and elastic laminar disruption are also two key cardiovascular manifestations of Marfan syndrome. As a connective tissue disorder with autosomal dominant inheritance, Marfan syndrome is caused by loss-of-function mutations in fibrillin-1, a matrix component of extracellular microfibrils [21]. Fibrillin-1 regulates activation of the cytokine TGFβ, and its deficiency results in enhanced TGFβ signaling [22], [23]. Recent studies have shown that abnormal activation of TGFβ contributes to the pathogenesis of Marfan syndrome [22]. Paradoxically, loss of function mutations in TGFβ receptor I or II also result in increased TGFβ signaling and give rise to Marfan syndrome [24], [25]. LRP1 is identical to the type V TGFβ receptor (TβR-V), which co-expresses with other TGFβ receptors (TβR-I, TβR-II and TβR-III) [13]. LRP1/TβR-V mediates TGFβ induced growth inhibition in concert with TβR-II/TβR-I/Smad2/3/4 signaling [13], [14] and TGFβ signaling is increased in LRP1 deficient mouse aortas [15].

**Figure 6:**
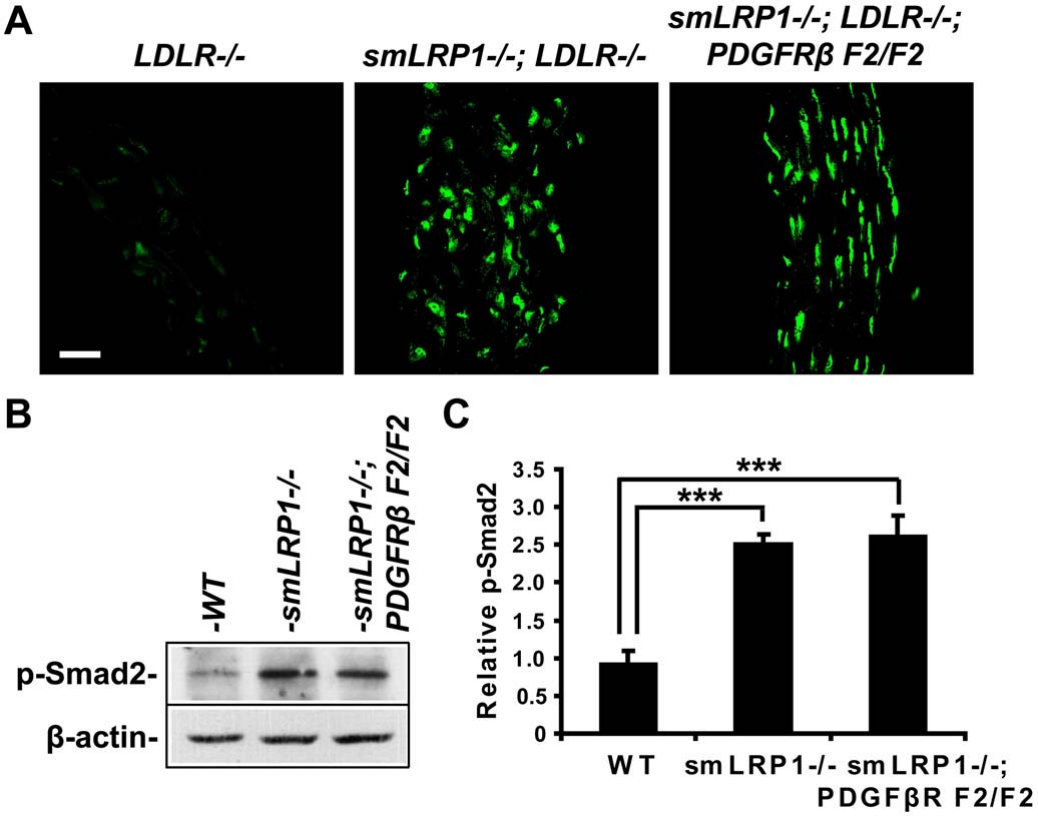
Increased Smad2 phosphorylation at Ser 465/467 in *smLRP1−/−; LDLR−/−* and *smLRP1−/−; LDLR−/−; PDGFRβ F2/F2* aortas. (A) Immunofluorescent staining of p-Smad2 (Ser 465/467, green) in aortas of atherosclerosis-free 1-month old mice. Scale bar, 20 µm. (B) Immunoblotting of aortic extracts for Smad2 phosphorylation at Ser 465/467. β-actin served as a loading control. (C) The density of p-Smad2 signals was normalized to the corresponding β-actin signals from the same blot and quantified using Image J software (NIH). Densitometric scanning from three independent experiments revealed a statistically significant average of 250%±13% in *smLRP1−/−* mice and 260%±29% in *smLRP1−/−; PDGFRβ F2/F2* mice, compared with wild type controls (92%±17%). Relative ratio of p-Smad2 in mouse aortas from the indicated genotypes was plotted. Data are presented as mean±SD. * *p*<0.001, n = 3.

To evaluate the TGFβ activation state in the aortas of the different genotypes, immunofluorescent staining and Western blotting of p-Smad2 were performed. Significantly increased Smad2 nuclear translocation was seen in *smLRP1−/−; LDLR−/−* aortas. Importantly, blockade of PI3K binding to PDGFRβ did not suppress over-activation of TGFβ signaling (Figure 6A). An approximately 2.5-fold increase of Smad2 phosphorylation at Ser 465/467 was also detected in LDLR-expressing, LRP1-deficient SMCs (Figure 6B, C). These data indicate that in the absence of LRP1, TGFβ signaling is abnormally activated independent of the absence or presence of the LDL receptor, and this over-activation of TGFβ is not suppressed by the PDGFRβ F2 mutation.

Taken together, our findings suggest that aorta elongation and elastic layer disruption, both hallmarks of Marfan syndrome, require the PDGFRβ dependent activation of PI3K. Activation of the TGFβ signaling pathway alone is not sufficient. Thus, LRP1 acts as a molecular switch that integrates TGFβ and PDGFRβ/PI3K signals and this is essential for maintaining the integrity of the vascular wall architecture.

## Discussion

In this study we have investigated the role of LRP1 for PI3K activation by PDGFRβ in SMCs, and the impact this LRP1 ‘checkpoint’ has for preventing atherosclerotic lesion formation and progression, as well as for the maintenance of vascular wall integrity. We found that the selective genetic blockade of PI3K activation by PDGFRβ substantially suppressed spontaneous atherosclerotic lesion development, which is prominent in *smLRP1−/−; LDLR−/−* mice. Furthermore, vascular wall elongation and medial thickening, due to SMC hyperproliferation, increased SMC migration and disruption of elastic layers are normalized throughout the entire aorta. Our findings suggest that PI3K is the main driving force that promotes SMC proliferation and migration, elastolysis, spontaneous atherosclerosis and lesion progression in the absence of LRP1.

Prominent atherosclerotic lesions preexisted in *smLRP1−/−; LDLR−/−* mice maintained on standard, low-fat and cholesterol-free rodent chow, but not in *smLRP1−/−* animals of comparable age. These data suggest that in the presence of an intact endothelium and low plasma cholesterol levels, proliferative signals alone are not sufficient to initiate the pathogenic mechanisms that culminate in classic atherosclerotic plaques. By contrast, the aorta of *LRP1+/+; LDLR−/−* mice appears histoanatomically normal despite increased plasma cholesterol levels on the same chow, and extensive atherosclerotic lesions develop only after feeding of a high-cholesterol diet for several months [26]. Thus, LRP1 in SMCs functions cell autonomously in the maintenance of vascular wall integrity and protection from cholesterol-induced atherosclerosis.

In the absence of smLRP1, the mouse aorta undergoes hyperplastic and hypertrophic changes that were apparent in young (7 weeks) as well as older (11 months old) mice indicating that they are not the result of aging, but the manifestation of an intrinsic change of smooth muscle phenotype. This is most likely caused by the increased expression and activation of PDGFRβ in smLRP1−/− mice and an accompanying increase in PI3K association with PDGFRβ. Disruption of an obligatory proatherogenic proliferative pathway, involving PI3K and PDGFRβ, prevents or greatly reduces lesion development at sites of high shear stress, such as the aortic arch and the abdominal aorta, where endothelial integrity is easily compromised. Thus, by selectively controlling SMC proliferation and migration independent of endothelial integrity and plasma cholesterol levels in a novel genetically complex animal model, we have been able to isolate and demonstrate the pivotal and interdependent roles of two central mechanisms of atherosclerotic lesion development.

Activation of the PDGFRβ results in actin reorganization in the forms of membrane ruffling and chemotaxis [11], [27], [28], [29], [30] and thus provides an excellent functional assay for the physiological activation of PDGFRβ through other genetic manipulations, such as the disruption of LRP1. PI3K binding to the cytoplasmic domain of activated PDGFRβ receptors requires phosphorylation at residues 739 and 750 and this interaction in turn activates the kinase [18], [31]. Replacement of these tyrosines by non-phosphorylatable phenylalanines prevents binding of PI3K and fails to mediate membrane ruffling and cell migration [28], [29]. As a result, the pronounced edge ruffling and circular membrane ruffling as well as greatly enhanced SMC migration that were observed in the absence of LRP1 were virtually normalized in mice in which PDGFRβ-dependent PI3K activation had been genetically disrupted. These findings show that the membrane ruffling and increased smooth muscle migration in *smLRP−/−* mice is critically dependent upon PI3K activation, which is mediated by PDGFRβ. Nevertheless, a caveat to this interpretation is that, although PI3K is the only known cellular signal transducer that interacts with pY739 and 750 of PDGFRβ, this do not exclude the possibility that another unknown signal modulator also interacts with this site and contributes to the pathogenic mechanism.

Marfan syndrome, a disorder of connective tissue architecture with prominent manifestations in the skeletal, ocular and cardiovascular systems, is caused by mutations in the fibrillin-1 gene [21], [32] or by loss of function mutations in TGFβ receptor I or II [24], [25]. TGFβ signaling is abnormally elevated in fibrillin-1-deficient mice [22], [23], [33] and human aortas [34] as well as TGFβ receptor I and II deficiency [24]. Previous data from our laboratory have shown nuclear accumulation of phosphorylated Smad2, an indicator of activation of TGFβ signaling, in the LRP1(TβR-V)-deficient vascular wall [15]. In the present study, we have reconfirmed these Marfan syndrome-like phenotypes, including elastic layer disruption, aorta elongation, and aneurysm formation in the presence of increased Smad2 phosphorylation when LRP1 is deficient in the SMCs. These phenotypic manifestations in the vascular wall were essentially abolished in *smLRP1−/−; PDGFRβ F2/F2* mice, however, the increased phosphorylation and nuclear translocation of Smad2 was not affected by the PDGFRβ mutations. These findings indicate that TGFβ activation through LRP1 precedes PDGFRβ-dependent PI3K signaling, and that activation of TGFβ signaling by itself is not sufficient to disrupt the vascular wall architecture. PDGFRβ-dependent PI3K activation appears to be necessary for the expression of the Marfan-like phenotypes. Suppression of PI3K activation by PDGFRβ prevents the Marfan-like phenotypic changes in the vascular wall in the presence of unabated TGFβ signaling, suggesting a pivotal role of LRP1-controlled and PDGFRβ-dependent PI3K activation in the pathogenesis of Marfan syndrome. Selective elimination of PDGFRβ-dependent PI3K activation thus could be a potential therapeutic target for both atherosclerosis and Marfan syndrome.

In conclusion, the current study reveals a novel PI3K-dependent mechanism by which LRP1 is essential for controlling the integrity of the vascular wall, and by which this multifunctional receptor potently protects against atherosclerosis and Marfan syndrome. The findings we have presented here shed new light on the molecular mechanisms that control cellular growth and migration, and which are thereby essential to the remodeling and repair of the vascular wall and for slowing or preventing degenerative disorders of the vascular wall.

## Materials and Methods

### Generation of the mouse strains

All experimental mice were maintained on a mixed C57BL/6/129 background. Transgenic mice expressing Cre recombinase specifically in smooth muscle cells (SM22 Cre) mated with *LRP1 loxP/loxP* mice to generate *smLRP1−/−* mice. Similarly, *smLRP1−/−; LDLR−/−* and *smLRP1−/−; LDLR−/−; PDGFRβ F2/F2* mouse strains were established. Paired littermates were utilized throughout the study.

### Animal & aorta preparation

Experiments were performed according to protocols approved by the Institutional Committee for Use and Care of Laboratory Animals. All animals were maintained on standard rodent chow (Teklad 6% fat) with water *ad libitum*. Mice were sacrificed and blood samples were collected for lipid analysis following a six-hour fasting period. Aortas were removed intact from the root of the aortic arch to the iliac bifurcation and preserved in 4% paraformaldehyde (PFA) for conventional morphological study.

### Oil Red O staining

Aortas were opened longitudinally under a dissecting microscope (Model Z30L, Cambridge Instruments). After fixation in 4% PFA, tissues were stained with 0.05% Oil Red O (s1848, Poly Scientific) at 60°C for 30 min. The aortas were then rinsed twice with 85% propylene glycol to develop the color.

**Hematoxylin-eosin (H&E), Masson's Trichrome and Hart's Elastin stainings** were performed according to established textbook methods [35], [36].

### Aortic extract preparation and Western blotting (WB)

The fat and connective tissue of the aorta were removed carefully. The aorta was homogenized in RIPA buffer with Proteinase Inhibitor Cocktail (P8430, Sigma) and Phosphatase Inhibitor Cocktail II (P5726, Sigma) to inhibit tyrosine protein phosphatases. After centrifugation at 20,000 xg for 30 minutes at 4°C, the supernatant was applied for Western blotting and the pellet was discarded.

Briefly, aortic extract was resolved on SDS-PAGE gel and transferred to nitrocellulose membranes (HybondTM-C Extra, RPN303 E, Amersham Biosciences). Blots were blocked with 5% skim milk, probed with the appropriate primary antibodies (α-PDGFRβ: #06-498, Upstate; α-p-Erk1/2: #9101, Cell Signaling; α-LRP1: 377, Herz Lab; α-TGFβRII: #06-227, Upstate; α-p-Smad2, #3108, Cell signaling) and then incubated with horseradish peroxidase-conjugated anti-rabbit secondary antibody (NA934V, Amersham Biosciences). Immunoreactive bands were visualized using an enhanced chemiluminescence Western blotting detection kit (RPN 2132, Amersham Biosciences).

### Co-immunoprecipitation

Aorta extracts were prepared as described above but using immunoprecipitation (IP) lysis buffer (50 mM Tris pH 7.4, 150 mM NaCl, 0.5% NP-40) instead of RIPA buffer.

Protein extract was pre-cleared with irrelevant non-immune serum. Nonspecific binding was precipitated with pre-swollen protein A-Agarose beads (P3476, Sigma). Supernatant was harvested by short spin and the appropriate specific primary antibodies were applied (α-PDGFRβ: #06-498, Upstate; α-p-Tyrosine: #05-321, Upstate). The suspension was probed at 4°C for 2 hours and then precipitated again with protein A-Agarose beads. The antigen-antibody-protein A Agarose complex was collected by a brief centrifugation, washed three times with a solution containing 50 mM Tris pH 7.4, 150 mM NaCl, 0.1% NP-40 and then three times with the same buffer without NP-40. The washed antigen-antibody-protein A Agarose complex was resolved on SDS-PAGE gel and immunoblotted with the relevant antibodies (α-p-Tyrosine: #05-321, Upstate; α-PI3K-p85: #06-496/#05-217, Upstate; α-PDGFRβ: #06-498, Upstate; α-LRP1: 377, Herz Lab; α-actin: A4700, Sigma).

### Primary SMC culture

Mouse primary SMC culture was established using the explant technique as previously described [37], [38]. All aortas were obtained from 8-week old male mice. Briefly, the aorta was dissected under sterile conditions, rinsed with PBS containing antibiotics, and the connective tissue and adventitia were removed carefully. The aorta was opened longitudinally and the intima was scraped on luminal surface. Then the aorta was minced into small pieces and placed into a T25 flask with high glucose (4.5 g/L) DMEM containing 15% FCS, 100 U/ml penicillin, 100 mg/ml streptomycin, 20 mM L-glutamine. Both explants and cells were cultured at 37°C in 5% carbon dioxide (CO_2_). Cells were detached by incubation with 0.25% trypsin-EDTA solution. Passages 5–15 were used in this study.

### Immunocytochemistry

Subcultured SMCs from the mouse aortic explants were allowed to grow on glass coverslips for 24 hours after trypsinization. The cells were fixed *in situ* with 95% ethanol, blocked with 5% non-immune goat serum, and probed with anti-α-smooth muscle actin (A2547, Sigma) and LRP1 (377, Herz Lab) antibodies. After three washes in PBS, the cells were incubated with Alexa Fluor 594 goat anti-mouse (A11032, Molecular Probes) and Alexa Fluor 488 goat anti-rabbit IgG antibodies (A11034, Molecular Probes). After three more washes in PBS, coverslips were mounted on glass slides using a DAPI-containing mounting medium (Vectashield^R^ Hard Set™, H-1500, Vector) and analyzed using a fluorescence microscope (Axioplan 2 Imaging, Carl Zeiss MicroImaging Inc.).

### Boyden chamber transmigration assay

SMC migration was measured using a 12-well modified Boyden chamber (AA12, Neuro Probe) hosting a polycarbonate filter with 8-µm pores (PFB8, Neuro Probe) as described [39]. 3×10^4^ cells in 100 µl were loaded into the top chamber of each well while the lower chambers were filled with SMC medium. After incubating at 37°C in 5% CO_2_ for 6 hours, non-migrated cells were scraped from the upper surface of the filter. Cells on the lower surface were fixed with 95% ethanol and stained with Harris Modified Hematoxylin (HHS-16, Sigma). The number of SMCs on the lower surface of the filter was determined by counting five continuous high-power (200×) fields of constant area per well. Experiments were performed three times in duplicate wells.

### Two-dimensional migration (scratch) assay

Subcultured 3×10^5^ SMCs were seeded into 60 mm Petri dishes. 10 µg/ml mitomycin C was applied to inhibit cell proliferation. Cells were incubated at 37°C in 5% CO_2_ overnight. The next day, part of the dish was denuded by scratching along a straight line. The dishes were put back to the incubator for 24 hours. The cells which appeared on the denuded area were treated as migrated cells. To quantify the migrated cells, the cells in five continuous high-power (200×) fields were counted and statistical analysis was performed.

### PDGF-BB chemotaxis assay

This experiment was performed as described above in the Boyden chamber transmigration assay with the exception that only 1×10^4^ cells were seeded. In addition, 10 ng/ml PDGF-BB was administrated to the lower chambers as an attractant.

### Immunohistochemistry

Aortas were isolated and fixed in 4% PFA for 1 hour. The tissues were sliced into 8 µm cross sections after embedded in OCT. The mounted sections were treated with 0.1% Triton X-100 for 5 minutes, blocked with 5% non-immune goat serum, and probed with anti-p-Smad2 (#3108, Cell signaling) rabbit antibody. After three washes in PBS, the sections were incubated with Alexa Fluor 488 goat anti-rabbit IgG antibodies (A11034, Molecular Probes). After three more washes in PBS, coverslips were mounted on glass slides with a DAPI-containing mounting medium (ProLong Gold antifade reagent with DAPI, P36935, Invitrogen).

### Statistical analysis

Statistical analyses were performed using two-tail Students' t-test. Results are given as mean±SD. A *p*<0.05 was considered significant.

## Supporting Information

Figure S1 Plasma Lipoprotein Profile. A significant increase of LDL-cholesterol was observed in LDLR−/− and smLRP1−/−; LDLR−/− mice. LRP1-deficiency in smooth muscle cells had no effect on the plasma lipoprotein profile.
(1.21 MB TIF)

Figure S2 Light microscopy of cryostat sections. Aortas of 7-week old mice were dissected out. 8 µm-thick cryostat sections were stained with DAPI. Aortic wall thickening was observed in smLRP1−/− mice.
(4.56 MB TIF)
